# Supplementary material for: Implantable bioelectronics for gut electrophysiology
Source: Nat Commun. 2025 Nov 20;16:10240. doi: 10.1038/s41467-025-65473-w (PMC12635400; doi:10.1038/s41467-025-65473-w)
Supplement: Supplementary file 2 — Description of Additional Supplementary Files [file 41467_2025_65473_MOESM2_ESM.pdf]

Description of Additional Supplementary Files

Supplementary Movie 1. Video of *in vivo* placement of implant under anesthesia. Video is looped to allow for examination for fine movements of tissue.

Supplementary Movie 2. Higher magnification of video of *in vivo* placement of implant under anesthesia. Video is looped to allow for examination for fine movements of tissue.

Supplementary Movie 3. Mechanical distension of ligated colon section through intraluminal saline injections. The implant conforms to the expansion of gut tissue.

Supplementary Movie 4. Freely-moving animal during chronic recording.

Supplementary Movie 5. Freely-moving rat, shortly after placement into open-field environment.
